# Small bowel cleanliness in capsule endoscopy: a case–control study using validated artificial intelligence algorithm

**DOI:** 10.1038/s41598-022-23181-1

**Published:** 2022-10-29

**Authors:** Dong Jun Oh, Youngbae Hwang, Ji Hyung Nam, Yun Jeong Lim

**Affiliations:** 1 Department of Internal Medicine, Dongguk University Ilsan Hospital, Dongguk University College of Medicine, 27 Dongguk-Ro, Ilsandong-Gu, Goyang, 10326 Republic of Korea; 2 Department of Electronics Engineering, Chungbuk National University, Cheongju, Republic of Korea

**Keywords:** Machine learning, Gastroenterology, Small intestine

## Abstract

Small bowel capsule endoscopy (SBCE) may need to be performed immediately after colonoscopy without additional bowel preparation if active small bowel diseases are suspected. However, it is unclear whether the small bowel cleanliness is adequately maintained even after SBCE is performed immediately after colonoscopy. We compared the small bowel cleanliness scores of the study group (SBCE immediately after colonoscopy) and control group (SBCE alone) using a validated artificial intelligence (AI) algorithm (cut-off score > 3.25 for adequate). Cases of SBCE in which polyethylene glycol was used were included retrospectively. Among 85 enrolled cases, 50 cases (58.8%) were the study group. The mean time from the last dose of purgative administration to SBCE was 6.86 ± 0.94 h in the study group and 3.00 ± 0.18 h in the control group. Seventy-five cases (88.2%) were adequate small bowel cleanliness, which was not different between the two groups. The mean small bowel cleanliness score for the study group was 3.970 ± 0.603, and for the control group was 3.937 ± 0.428. In the study group, better colon preparation resulted in a higher small bowel cleanliness score (p = 0.015). Small bowel cleanliness was also adequately maintained in SBCE immediately after colonoscopy. There was no difference between the time and volume of purgative administration and small bowel cleanliness.

## Introduction

Small bowel capsule endoscopy (SBCE) is currently the key modality for diagnosing various SB diseases, such as obscure gastrointestinal (GI) bleeding, known or suspected SB Crohn’s disease without stenosis, small bowel tumours or polyposis, and refractory celiac disease^1–3^. Unlike conventional endoscopy, bowel cleansing cannot be actively performed during the SBCE examination. So proper bowel preparation before SBCE is important to improve small bowel mucosal visualization^4^.

For bowel preparation before colonoscopy, the European Society of Gastrointestinal Endoscopy (ESGE) recommends split-dose preparation or same-day preparation for elective colonoscopy. ESGE also recommends that the last dose of purgative be taken within 5 h before the colonoscopy and completed 2 h before the colonoscopy^5^. The American Society for Gastrointestinal Endoscopy (ASGE) recommends that the second dose of purgative administration be started 3 to 8 h before the endoscopy^6^. However, the consensus on the timing and method of bowel preparation for SBCE is still controversial^7^.

If SB bleeding is suspected after upper endoscopy and colonoscopy in patients with non-severe GI bleeding, SBCE is performed^1–3,8^. In this case, it is recommended to perform SBCE as soon as possible to increase the diagnostic yield of SB bleeding^4,9^. SBCE should also be considered when non-obstructive SB Crohn’s disease is suspected on colonoscopy^10^. SBCE may need to be performed immediately after colonoscopy. It was also known that poor quality of SBCE images has a significant negative effect on the diagnosis of SB pathology, especially malignancy^11^.

However, there is a lack of research on whether SB cleanliness is adequate when SBCE is performed immediately after colonoscopy. If the SB cleanliness is significantly poor in SBCE immediately after a colonoscopy, additional preparation may be required after colonoscopy and before SBCE. Nevertheless, additional purgative administration or fasting for SBCE is also a burden on the patient and there is a risk of delayed diagnosis. Thus, it is necessary to compare whether there is a difference in SB cleanliness between SBCE immediately after colonoscopy and SBCE alone.

Recently, a study using an artificial intelligence (AI) algorithm trained by PillCam (GIVEN Imaging Ltd., Yoqneam, Israel) images was conducted. This validated AI algorithm calculated an objective and automated SB cleanliness score for the full-length SB images^12^. In this study, we used a validated AI algorithm and compared SB cleanliness scores between SBCE immediately after colonoscopy and SBCE alone. So, we decided to identify whether colonoscopy before SBCE affected the difference in SB cleanliness scores.

## Results

### Baseline characteristics

Out of 100 cases satisfying the inclusion criteria, 85 subjects were finally enrolled. The mean age of the patients who underwent SBCE was 49.2 years, and 60% were male. The main reasons for SBCE were suspected SB bleeding (45.9%) and Crohn’s disease (43.5%). SBCE immediately after colonoscopy was performed in 58.8%; sedation colonoscopy with intravenous midazolam and pethidine was performed in all cases. The mean gastric transit time was 1.12 ± 1.16 h, and the mean SB transit time was 5.64 ± 2.35 h. The mean gap from the start of the last dose of purgative administration to SBCE was 5.27 ± 2.04 h. The mean gap in SBCE immediately after colonoscopy was 6.86 ± 0.94 h, and the mean gap in SBCE alone was 3.00 ± 0.18 h (Table 1).

**Table 1:** Baseline characteristics of patients who underwent small bowel capsule endoscopy (SBCE) (N = 85).

Variables	SBCE after colonoscopy (n = 50)	SBCE alone (n = 35)	p
**Mean age, years**	49.1 ± 19.8	49.0 ± 23.0	0.981
> 65 years old	10 (20.0%)	9 (25.7%)	0.534
**Male gender**	35 (70.0%)	17 (48.6%)	0.046
ASA			
I–II	43 (86.0%)	28 (80.0%)	0.463
III–IV	7 (14.0%)	7 (20.0%)	
**Reasons for SBCE**			
(Suspected) small bowel bleeding	23 (46.0%)	15 (42.9%)	0.838
(Suspected) Crohn’s disease	22 (44.0%)	17 (48.6%)	
(Suspected) tumor or polyposis	4 (8.0%)	3 (8.5%)	
(Suspected) drug induced enteropathy	1 (2.0%)	0 (0.0%)	
**Diagnostic yield**			
Small bowel bleeding	43.5% (10/23)	53.3% (8/15)	0.552
Crohn’s disease	63.6% (14/22)	64.7% (11/17)	0.945
Tumor or polyposis	50% (2/4)	33.0% (1/3)	0.659
Drug induced enteropathy	100% (1/1)		N/A
Taking prokinetics after SBCE	32 (64.0%)	10 (28.6%)	0.001
Mean small bowel transit time, hours	5.85 ± 2.08	5.36 ± 2.69	0.353
Mean gastric transit time, hours	1.32 ± 1.18	0.84 ± 1.10	0.059
Mean time from purgative* to SBCE, hours	6.86 ± 0.94	3.00 ± 0.18	0.000

*ASA* American Society of Anesthesiologists, *SD* standard deviation.

*Last dose of purgative.

### Small bowel cleanliness scores calculated by the AI algorithm

**Figure 1:**
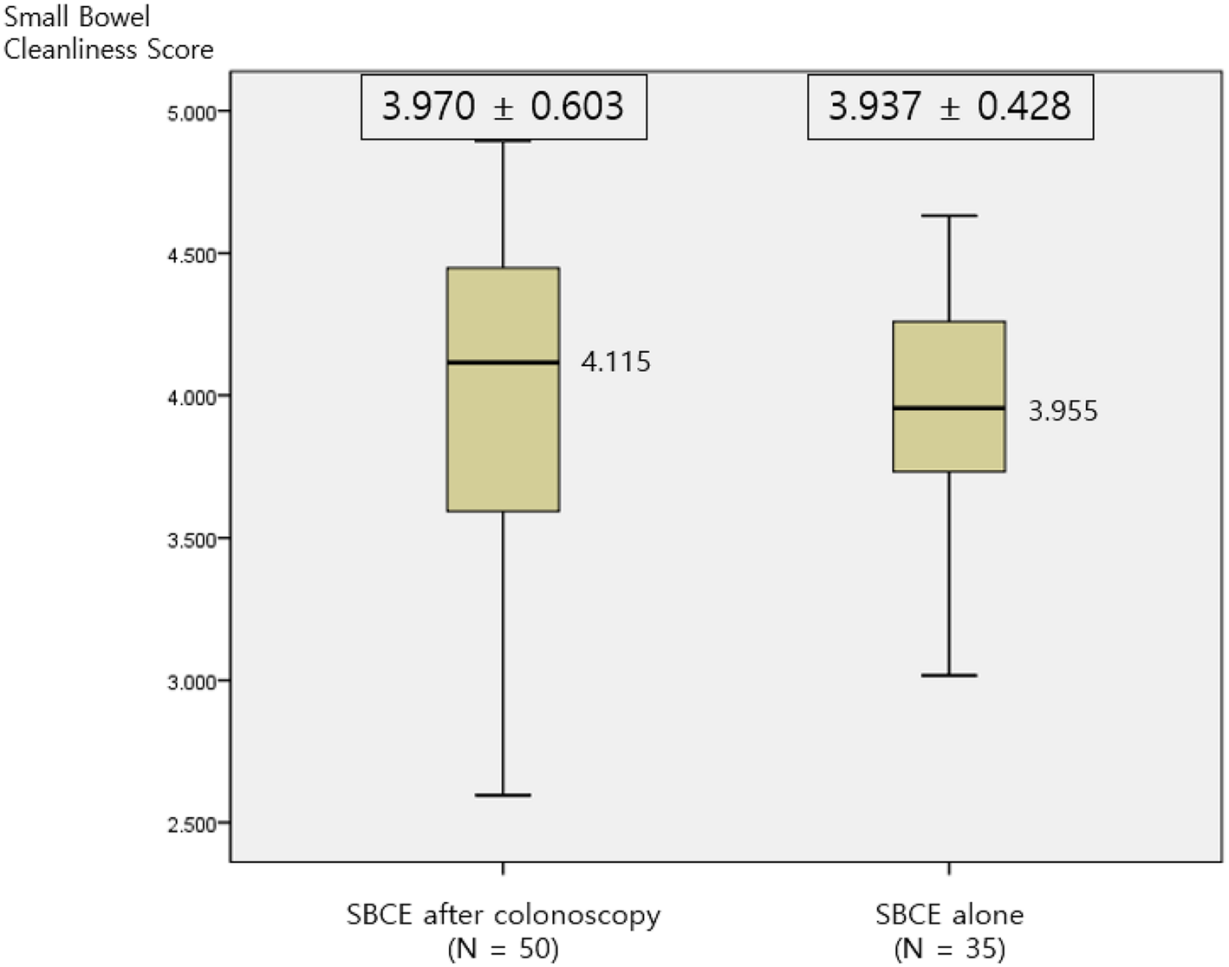
Relationship between small bowel cleanliness score and pre-colonoscopy. The mean small bowel cleanliness score in small bowel capsule endoscopy (SBCE) immediately after colonoscopy was 3.970 ± 0.603, and in SBCE alone was 3.937 ± 0.428. There was no significant difference between the colonoscopy and small bowel cleanliness score (p = 0.785).

The mean number of full-length images in SBCE was 12,068.8 ± 6597.5. The mean cleanliness score of all SBCE cases calculated by the AI algorithm was 3.956 ± 0.535. The mean score in the group with SBCE immediately after colonoscopy was 3.970 ± 0.603 and the mean score in the group with SBCE alone was 3.937 ± 0.428. There was no significant difference between the two groups (p = 0.785) (Fig. 1). Of the all SBCE cases, 88.2% showed adequate mucosal visualization (score > 3.25), and 50.6% showed good mucosal visualization (score > 4.0) (Table 2).

**Table 2:** Analysis of artificial intelligence algorithm calculated full length small bowel capsule endoscopy (N = 85).

Variables	SBCE after colonoscopy (n = 50)	SBCE alone (n = 35)	p
Mean number of images in SBCE (SD)	12,976.6 ± 7240.3	10,771.9 ± 5389.3	0.130
AI calculated Cleanliness score (SD)	3.970 ± 0.603	3.937 ± 0.428	0.785
Cases of adequate cleansing	43 (86.0%)	32 (91.4%)	0.445
Cases of good cleansing	27 (54.0%)	17 (48.6%)	0.622

*SBCE* small bowel capsule endoscopy, *AI* artificial intelligence, *SD* standard deviation.

In univariate analysis, males and delayed gastric transit time were associated with inadequate cleanliness. But no statistical significance was observed in multivariate analysis. There was no statistical significance between other variables and adequate cleanliness score (Table 3).

**Table 3:** Univariate and multivariate analysis of adequate cleansing cases (N = 85).

Variables	Cleanliness score		Univariate, p	Multivariate p (OR; 95% Cl)
	Adequate (n = 75)	Inadequate (n = 10)		
Age > 65 years	17 (22.7%)	2 (20.0%)	0.849	
Male gender (%)	43 (57.3%)	9 (90.0%)	0.046	0.096 (6.33;0.72–55.46)
ASA class I or II (%)	63 (84.0%)	8 (80.0%)	0.749	
ASA class ≥ III (%)	12 (16.0%)	2 (20.0%)		
**Lesions identified in SBCE**				
Bleeding/vascular lesions	11 (14.7%)	3 (30.0%)	0.592	
Ulcers/erosions	35 (46.7%)	4 (40.0%)		
Polyps	4 (5.3%)	0 (0.0%)		
No definite lesion	25 (33.3%)	3 (30.0%)		
**Diagnostic yield**				
Small bowel bleeding	42.4%	80.0%	0.117	
Crohn’s disease	65.7%	50.0%	0.535	
Tumor or polyposis	50.0%	0.0% (0/1)	0.350	
Drug induced enteropathy	100%		N/A	
Pre-colonoscopy (all sedation)	43 (57.3%)	7 (70.0%)	0.445	0.729 (1.38;0.22–8.68)
Prokinetics	37 (49.3%)	5 (50.0%)	0.968	
Mean time from purgative* to SBCE	313.8 ± 123.7	335.4 ± 118.1	0.604	
Prolonged time to SBCE (> 7 h)	20 (26.7%)	4 (40.0%)	0.379	0.530 (0.58;0.11–3.17)
Mean small bowel transit time, min	340.3 ± 143.3	327.8 ± 126.6	0.795	
**Mean gastric transit time, min**	61.6 ± 64.1	108.6 ± 97.8	0.045	
Delayed gastric transit (> 90 min)	16 (21.3%)	5 (50.0%)	0.048	0.107 (0.30;0.07–1.29)

*ASA* American Society of Anesthesiologists, *SD* standard deviation.

*Last dose of purgative.

### Purgative administration time and small bowel cleanliness scores

**Figure 2:**
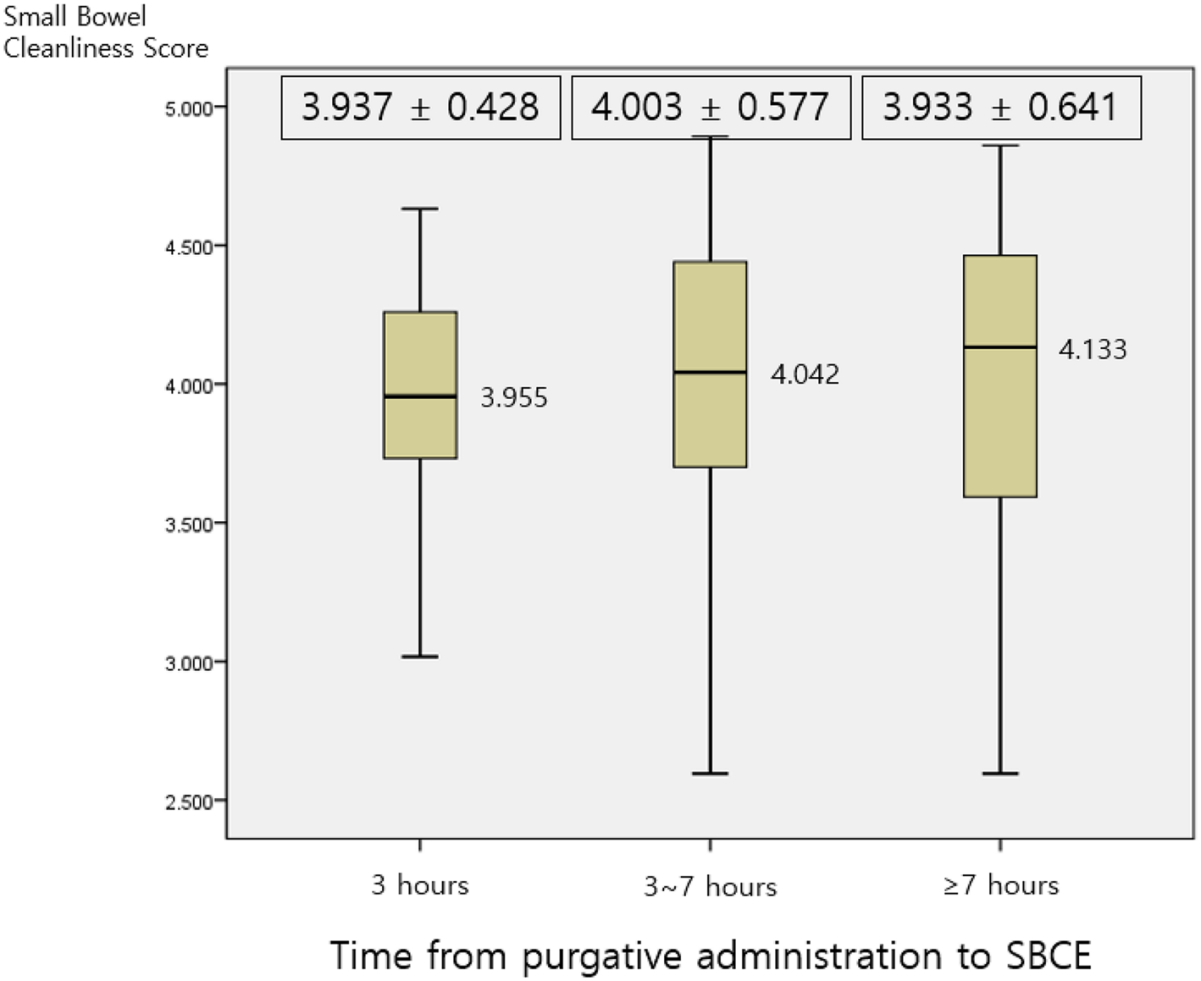
Relationship between small bowel cleanliness score and time from the last dose of purgative administration to small bowel capsule endoscopy (SBCE). The time from the last dose of purgative administration to SBCE was divided into 3 h and 7 h intervals. There was no significant difference between the three groups (p = 0.831).

The time from the last dose of purgative administration to SBCE was divided into 3 hoursand 7 h intervals. The mean small bowel cleanliness score in 3 h intervals was 3.937 ± 0.428, and between 3 and 7 h was 4.003 ± 0.577, and > 7 h was 3.933 ± 0.641 (Fig. 2). There was no significant difference between the three groups (p = 0.831).

### Association with colon preparation scale and small bowel cleanliness scores

**Figure 3:**
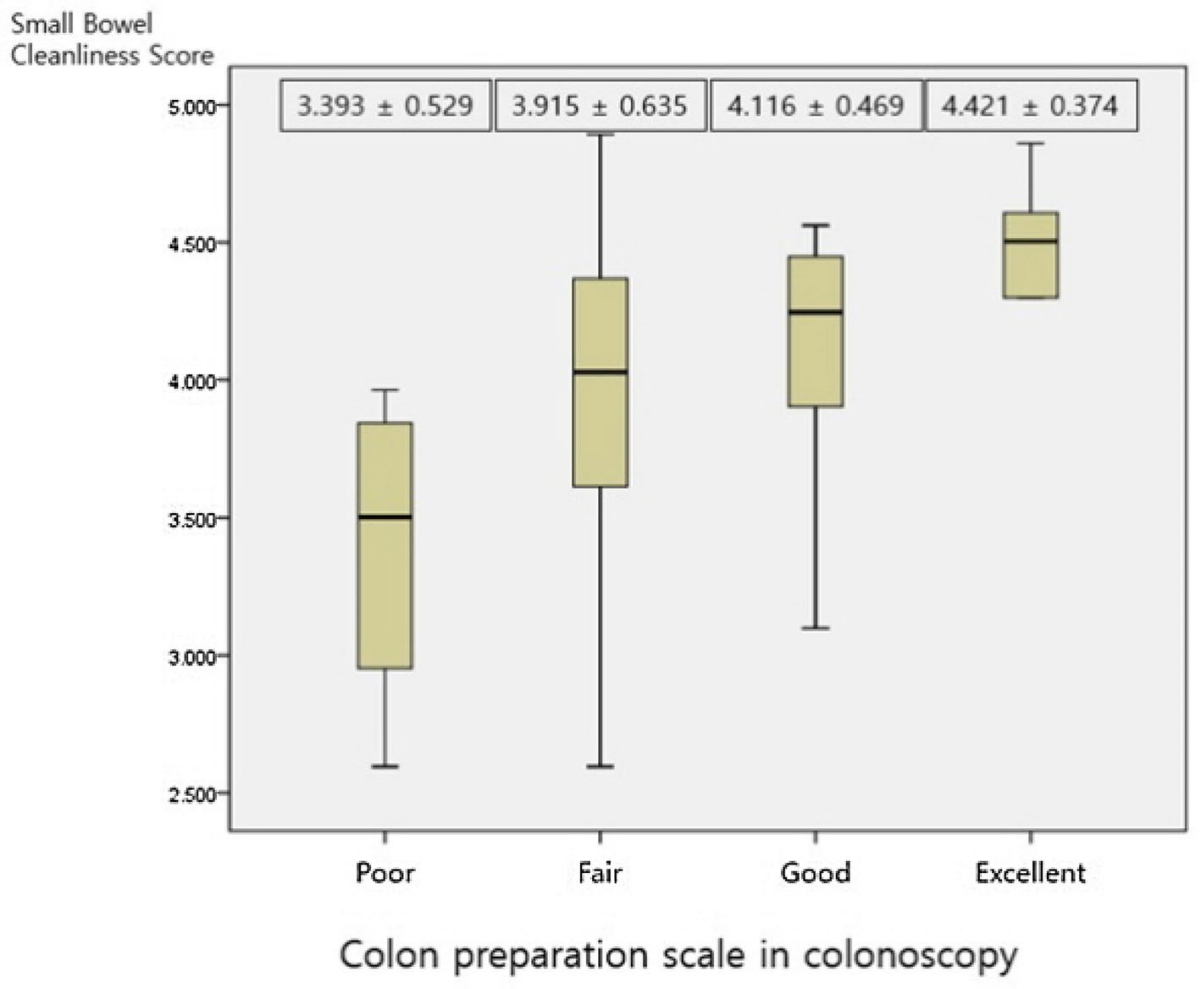
Colon preparation scale in colonoscopy and small bowel cleanliness scores. The colon preparation scale was measured by Aronchick Scale (from inadequate to excellent). The colon preparation was better, the small bowel cleanliness score was higher (p = 0.015).

In the case of performing SBCE immediately after a colonoscopy, the colon preparation scale was measured by Aronchick Scale (inadequate ~ excellent)^13^. Poor preparation was confirmed in 6 cases, fair preparation in 24 cases, good preparation in 14 cases, excellent preparation in 6 cases, and no inadequate preparation. The mean small bowel cleanliness score in poor preparation was 3.393 ± 0.529, fair preparation was 3.915 ± 0.635, good preparation was 4.116 ± 0.469, and excellent preparation was 4.421 ± 0.374 (p = 0.015) (Fig. 3).

### Diagnostic yield according to pre-colonoscopy and small bowel cleanliness scores

In the case of performing SBCE immediately after a colonoscopy, the diagnostic yield for small bowel bleeding was 43.5%, 63.6% for Crohn’s disease, 50% for tumour or polyposis, and 100% for drug-induced enteropathy. There was no statistically significant difference when compared with SBCE alone (53.3% for small bowel bleeding, 64.7% for Crohn’s disease, 33.0% for tumour or polyposis). (Table 1).

When the diagnostic yields were compared with the adequate and inappropriate cleansing groups, small bowel bleeding was 42.4% vs. 80%, Crohn’s disease 65.7% and 50.0% and tumour or polyposis 50.0% vs. 0.0%. There was also no statistically significant difference. (Table 3).

### Safety of taking capsule endoscope after sedative colonoscopy

To fully wake up from sedation, antidotes such as flumazenil and naloxone were administered, and sufficient recovery time was allowed. Also, before taking the capsule endoscope, it was confirmed that there was no problem swallowing water. No adverse events occurred when the capsule endoscope was taken after sedation colonoscopy in this study. In addition, no SBCE-related complications such as capsule retention and bowel obstruction were observed.

## Discussion

In this study, using a validated AI algorithm, we confirmed that small bowel cleanliness was adequately maintained in SBCE immediately after a colonoscopy, similar to that in SBCE alone. Also, there was no difference between the time and volume of purgative administration and small bowel cleanliness. However, in poor colon preparation, SB cleanliness score was not as good as in fair, good and excellent colon preparation. So, if the colon preparation is good, SBCE can be performed immediately after colonoscopy, but, if colon preparation is not good, additional bowel preparation before SBCE may be necessary.

In the case of SBCE immediately after a colonoscopy, we expected poor bowel preparation due to air insufflation during colonoscopy and the digestive enzymes constantly secreted by the small bowel. However, there was no relationship between SBCE immediately after colonoscopy and small bowel cleanliness.

Adequate bowel cleanliness is one of the critical factors for the performance and diagnostic capacity of SBCE^4,11^. However, since small bowel cleanliness is subjective and takes a long time to be measured by clinicians, intra-observer variation is inevitable^14^. For this reason, several studies have been conducted to identify the cleanliness of SBCE using an AI algorithm objectively^12,15–17^. We confirmed the objective SB bowel cleanliness scores by using a validated AI algorithm that was trained using SBCE images^12^.

Whether to take a purgative before SBCE, what type, and the ideal time to take it are still controversies^4,18^. First, there is controversy about the effectiveness of purgative preparations. In a meta-analysis study comparing fasting with clear liquid intake only and purgative administration, there was no significant difference between the two preparation methods in diagnostic yield and mucosal visualization^19^. Also, two randomized clinical trials (RCTs) reported that there was no difference in mucosal visualization when clear liquids and purgatives were compared^20,21^. However, a recent multi-center RCT reported that taking one liter of PEG on the same day of the SBCE examination resulted in superior SB mucosal visualization compared to the fasting alone group (66.3% for one liter of PEG vs. 32.5% for fasting)^22^. A Korean study that analyzed nationwide data found that 70% of the patients with SBCE had taken two liters of PEG and ascorbic acid since 2014. But there was no statistical significance in the comparison of adequate bowel preparation between the two liters of PEG and the fasting alone. (68.3% for two liters of PEG vs. 70.4% for fasting)^23^. Regarding the purgative administration time, one article mentioned that it should be taken 12 h before SBCE^24^. However, a recent multicenter study reported that low-dose PEG administration four hours before SBCE, which was similar to the protocol in our hospital, was ideal^22^.

Although there was no significant difference between purgative interval and small bowel cleanliness score, a significant correlation was confirmed with colon preparation scale and small bowel cleanliness score. Even in poor colon preparation, the mean small bowel cleanliness score (3.393 ± 0.529) was higher than 3.25, which is the cut-off value of adequate cleanliness. But as the colon preparation was better, the small bowel cleanliness score was higher (p = 0.015). Therefore, in case of poor colon preparation, additional purgative administration before SBCE should be considered.

In terms of diagnostic yield, there was no significant difference with whether pre-colonoscopy was performed or not. However, the diagnostic yield of small bowel bleeding was slightly higher in the inadequate cleansing group than in the adequate cleansing group (80% vs. 42.4%, p = 0.117). It was determined that the AI algorithm measured the low cleanliness score in the case of active bleeding because mucosal visualization was lowered by blood. Therefore, in the case of a low small bowel cleanliness score, it may be possible to estimate the possibility of active bleeding.

Our study had several limitations. First, this study was a retrospective and single-center study. As a result, it is possible that several variables were not controlled. In addition, although bowel preparation education was provided to the patients uniformly, the preparation may have been inadequate depending upon the patient. Finally, because the number of inadequate cleansing groups was small, it was difficult to compare the diagnostic yields accurately. Additional large-scale studies are needed to compare diagnostic yields.

In conclusion, our study using a validated AI algorithm at the full-length small bowel level identified no significant effect on bowel cleanliness and diagnostic yield even if SBCE was performed immediately after colonoscopy. Small bowel cleanliness was not significantly related to the dose and time of purgative taken before SBCE. Also, adequate mucosal visualization was maintained even when same-day and low-dose purgative preparation was used before SBCE. However, additional preparation before SBCE may be necessary if colon preparation is not good. A further well-designed prospective study is needed.

## Methods

### Study population and variables

We performed a retrospective, single-center study at Dongguk University Ilsan Hospital, Republic of Korea. Patients who underwent SBCE (PillCam SB3, GIVEN Imaging Ltd., Yoqneam, Israel) between January 1, 2018, and December 31, 2020, were included in the study. Patients who underwent SBCE other than PillCam SB3, incomplete (unable to identify the cecum) SBCE studies, and those with inaccurate or missed data were excluded.

Eighty-five patients who met the inclusion criteria were finally enrolled in the study. The following variables were analyzed based on electronic medical records and endoscopic images: age, gender, American Society of Anesthesiologists (ASA) classification, whether colonoscopy was performed before SBCE, reasons for SBCE, time from taking the last dose of purgative to colonoscopy and SBCE, whether prokinetics were used, gastric and small bowel transit time. This study was approved by the Institutional Review Board of Dongguk University Ilsan Hospital (DUIH 2021-08-036-001). All methods in this study were carried out in accordance with relevant guidelines and regulations. And, informed consent was obtained from all subjects or, if subjects are under 18, from a parent and/or legal guardian.

### Endoscopy procedure and purgative administration time (Fig. 4)

**Figure 4:**
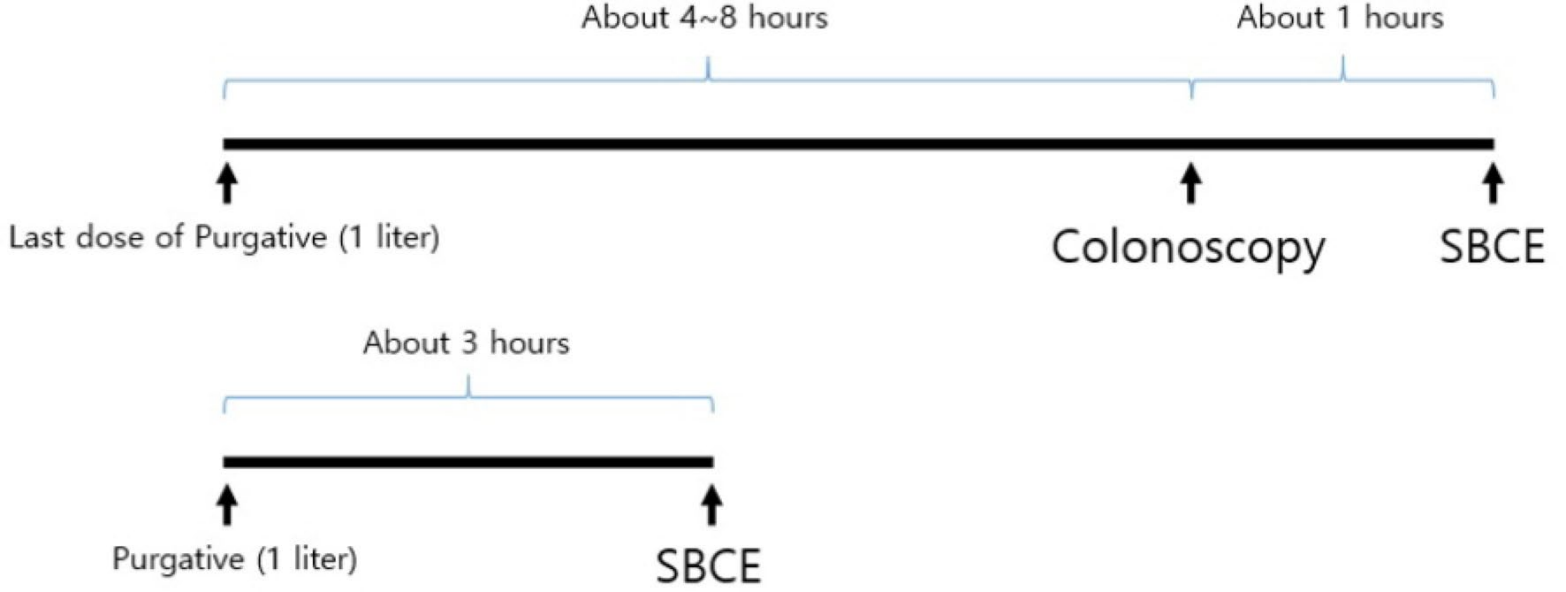
Schematic of the study process. (A) In the case of performing a small bowel capsule endoscopy (SBCE) immediately after colonoscopy, bowel preparation was performed by the split-dose or same-day regimen using 2 L of polyethylen glycol (PEG) + ascorbic acid. After colonoscopy, SBCE was performed about an hour later if necessary. (B) The bowel preparation for SBCE was performed by same-day and low dose preparation. Patients undergoing SBCE were instructed to start taking the 1 L of purgative 3 h before the SBCE, and finish taking it 1 h before the SBCE.

#### Small bowel capsule endoscopy alone

Patients undergoing only SBCE were instructed to fast for 12 h overnight, start taking the purgative 3 h before the SBCE, and finish taking it 1 h before the SBCE. All patients were instructed to take a purgative consisting of half dose (total 1 L with clear liquid) of polyethylen glycol (PEG) 3350 + ascorbic acid (Coolprep™, Taejoon Pharm Co., Ltd, Seoul, Korea).

#### Small bowel capsule endoscopy after colonoscopy

In this case, SBCE was performed about 1 hour after the end of the colonoscopy. For the colonoscopy, the colon was prepared by full dose (total 2 liter with clear liquid) of Coolprep^TM^ using either the split-dose or the same-day regimen. In the case of colonoscopies before noon, the patients were instructed to take the first dose at 8 p.m. the day before and the last dose at 6 a.m. on the day of colonoscopy as in the split-dose regimen. In the case of colonoscopies in the afternoon, the patients were instructed to take the first dose at 6 a.m. and the last dose at 9 a.m. on the day of colonoscopy as in the same-day regimen. The patients were instructed to complete taking the purgative 2 hours before the colonoscopy. Midazolam and pethidine were administered for sedation colonoscopy. If the colonoscopy was completed and the indications for SBCE were applicable, antidotes such as flumazenil and naloxone were administered and the patient was observed for one hour. And then, capsule endoscope was swallowed under the supervision of a medical staff. A prokinetic agent was administered if necessary when slow gastric transit time was expected in relation to sedative administration. If there was a large amount of SB bleeding or if SB stricture was suspected, device-assisted enteroscopy or abdomen & pelvis computed tomography was performed before SBCE. These cases were excluded from this study.

### Artificial intelligence (AI) algorithm for calculating bowel cleanliness score

In this study, a convolutional neural network (CNN) algorithm for calculating bowel cleanliness scores based on InceptionResnetV2 was used^11^ CNN algorithm is a subset of AI algorithm and consists of several convolutional layers and pooling layers. In each convolutional layer, different features of the image are extracted to identify the image. In our CNN algorithm, each SBCE image was calculated according to a 5-step scoring method. A cleanliness score of one was calculated when the mucosal visualization was less than 25% and a score of 5 when the mucosal visualization was 90% or more. In the previous study, when the cut-off cleanliness score value was 3.25, the area under the curve (AUC) of 0.977 for adequate and inadequate bowel preparation was confirmed. Therefore, the same cut-off score (3.25) was applied in this study as well.

**Figure 5:**
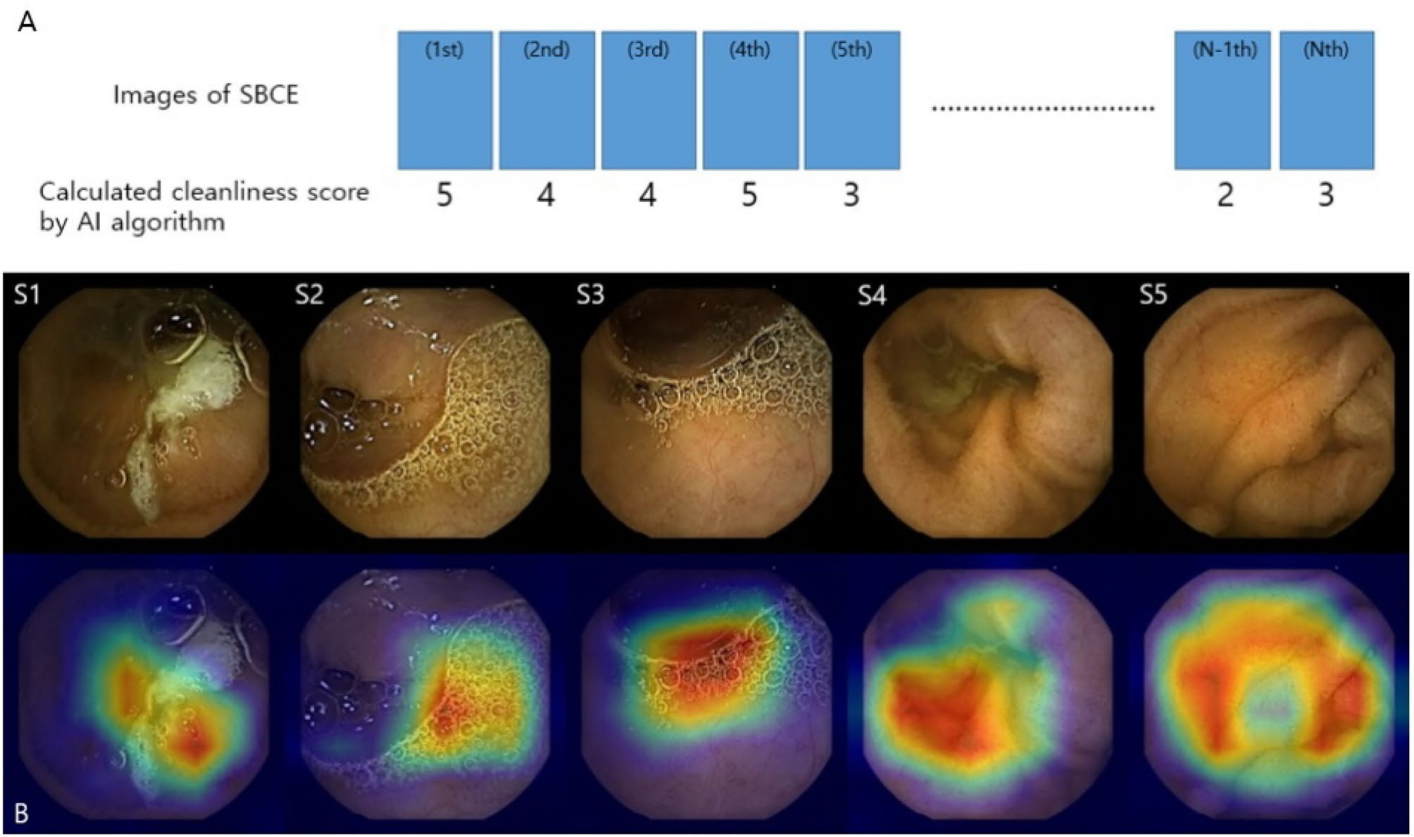
Real example of small bowel cleanliness score calculated by the artificial intelligence (AI) algorithm in a small bowel capsule endoscopy case (Case No. 60). (**A**) The small bowel cleanliness was scored from 1.0 (S1) to 5.0 (S5). (**B**) The image analysis by AI algorithm was expressed in a Grad-CAM visualization (heat-map).

Two expert endoscopists (Oh, D.J., Nam, J.H.) identified the images of duodenal and cecal transition in each SBCE case. Afterwards, only small bowel images were extracted and a small bowel cleanliness score was automatically calculated by the AI algorithm that measures the score of each image (Fig. 5).

### Outcomes and statistical analyses

The primary outcome was the difference in small bowel cleanliness scores between the SBCE immediate after colonoscopy and SBCE alone. The secondary outcome was to identify the factors related to poor small bowel cleanliness scores. Student’s *t* test and Chi-square analysis were performed on the variables. Analysis of variance (ANOVA) were performed to analyze the differences among the three or more groups. Factors for poor preparation were analyzed by logistic regression with odds ratios (ORs) and 95% confidence intervals (CIs). Statistical significance was set at a p value of < 0.05 in both univariate and multivariate analyses. Statistical analysis was carried out by IBM SPSS Statistics v25.

## Data Availability

The data that supports the findings of this study are available within the article.
